# Effect of a Drug-Eluting Stent vs. Bare Metal Stent for the Treatment of Symptomatic Intracranial and Vertebral Artery Stenosis

**DOI:** 10.3389/fneur.2022.854226

**Published:** 2022-07-13

**Authors:** Jiang-Hua Si, Ning Ma, Feng Gao, Da-Peng Mo, Gang Luo, Zhong-Rong Miao

**Affiliations:** Department of Interventional Neuroradiology, Beijing Tiantan Hospital, Capital Medical University, Beijing, China

**Keywords:** cerebrovascular disease, stroke, endovascular treatment, in-stent restenosis, drug-eluting stent, bare-metal stent

## Abstract

**Background:**

For patients with symptomatic intracranial and vertebral artery stenosis who receive endovascular treatment, in-stent restenosis (ISR) is associated with the recurrence of ischemic stroke. This study evaluated a drug-eluting stent (DES) vs. bare metal stent (BMS) for the treatment of symptomatic intracranial and vertebral artery stenosis.

**Methods:**

The trial was a multicenter, 1:1 randomized, prospective feasibility clinical trial with 10 participating centers in China from March 2014 to October 2015. Eligible patients had symptomatic intracranial and vertebral artery stenosis (70%−99%) and had medical drug treatment failure. The primary endpoint was the rate of in-stent restenosis at 180 days of randomization. The secondary endpoint was a composite of the following two outcomes: (1) ischemic stroke or transient cerebral ischemia (TIA) in the same territory as the presenting event (distal to the target lesion) between 30 days and 1 year after randomization and (2) successful stent implantation. The safety outcome was the presence of stroke in any territory and death within 30 days of randomization or adverse events. Group *t*-tests or Wilcoxon rank-sum tests were used for the intergroup comparison of quantitative data according to the data distribution. The chi-square test or exact probability method was used for the classification data. The Wilcoxon rank-sum test or CMH test was used for the categorical data.

**Results:**

We enrolled 188 patients at 10 medical centers in China (92 assigned to the DES group and 96 to the BMS group). The mean age of the 188 study participants was 61.6 years (range, 38–75 years); 152 participants (80.9%) were male. There were 28 patients (43.8%) with an ISR at 180 days in the BMS group and 10 patients (14.5%) in the DES group [risk difference, 29.3% (95% CI, 14.5%−44.0%); *P* = 0.001]. The percent of patients with ischemic stroke or TIA in the same territory between 30 days and 1 year was 5.2% (5/96) in the BMS group and 2.2% (2/92) in the DES group [risk difference, 3.0%; (95% CI, −2.3% to 8.2%); *P* = 0.354]. The percent of patients with successful stent implantation was 99.0% (95/96) in the BMS group and 97.8% (90/92) in the DES group [risk difference, 1.1%; (95% CI, −1.7% to 3.9%); *P* = 0.584]. In total, five patients (5.2%) in the BMS group and three patients (3.3%) in the DES group [risk difference, 1.9%; (95% CI, −2.3% to 6.1%); *P* = 0.721] had stroke in any territory and death within the 30-day follow-up. Total adverse events occurred 167 times in 72 patients (75.0%) in the BMS group compared with 114 times in 59 patients (64.1%) in the DES group [risk difference, 10.9%; (95% CI, −0.1% to 21.7%); *P* = 0.115].

**Conclusions:**

Among patients with symptomatic intracranial arterial stenosis and vertebral artery stenosis, the use of a drug-eluting stent compared with a bare metal stent resulted in a decreased risk of ISR, similar successful stent implantation, and similar adverse events. These findings support the use of a drug-eluting stent for patients with symptomatic intracranial arterial stenosis and vertebral artery stenosis.

**Clinical Trial Registration:**

http://www.chictr.org.cn/showproj.aspx?proj=148272, identifier: ChiCTR2200055925.

## Introduction

Stenting is an important treatment for the prevention and treatment of ischemic stroke, but operation-related complications limit the application of stenting in the diagnosis and treatment of ischemic stroke. In-stent restenosis (ISR) after stenting is associated with several common complications, especially in intracranial and vertebral arteries. A systematic retrospective study included 1,177 patients with intracranial atherosclerotic lesions in 31 studies (1). The results showed that the incidence of ISR after balloon expandable stenting was lower than that in the self-expandable stent group (13.8 vs. 17.4%). Some studies have reported that the incidence of ISR after bare-metal stents (BMS) of the extracranial segment of the vertebral artery is higher than that of intracranial arteries, and it is up to 20.6%−33.6% (2–4).

Drug-eluting stents (DESs) are equipped with anti-vascular endothelial cell proliferation drugs on their surface or inside of them. Compared with bare metal stents (BMSs), the slow release of drugs can inhibit the proliferation and migration of vascular smooth muscle cells in the stent, inhibit intravascular thrombosis, and prevent restenosis. At present, DES is used in the treatment of symptomatic intracranial artery and extracranial vertebral artery lesions. There are few studies on the application of DESs in ischemic stroke. Some clinical trials have observed that DES has a low restenosis rate when compared with BMS (2, 3, 5–9). However, most of these studies are small-sample, short-term follow-up, and descriptive trials, and there is a lack of large-sample, long-term follow-up, and randomized controlled trials.

In this trial, we examined percutaneous transluminal balloon angioplasty with stenting (DES vs. BMS) in symptomatic intracranial and extracranial vertebral stenosis. The trial was a multicenter randomized study designed to evaluate the safety and effectiveness of balloon-expandable DES in patients with cerebral or retinal ischemia attributed to intracranial and extracranial vertebral stenosis. Here, we report the final trial results.

## Materials and Methods

### Study Design and Objectives

This trial was a randomized multicenter clinical trial with 10 participating centers in China from March 2014 to October 2015. Approval by each site's institutional review board or Ethics Committee was obtained. Written informed consent was obtained from the patient or his or her legally authorized representative. Race and ethnicity were self-reported.

### Patient Population

The executive committee established the inclusion and exclusion criteria. Patients who were considered for inclusion in the study were 18–75 years of age, had symptomatic intracranial and vertebral artery stenosis (70%−99%) involving the internal carotid, middle cerebral, intracranial vertebral, or basilar arteries, and had medical drug treatment failure. Medical treatment failure was defined as the use of at least one antithrombotic drug and the presence of positive risk factors at the time of stroke or TIA intervention. Symptomatic stenosis was defined as a stroke or a TIA within 90 days, and the stroke or TIA had to be attributed to the responsible lesion that was treated in this study. The presence of stenosis was initially determined by TCD/MRA/CTA and was then confirmed by DSA. The target lesion length was ≤ 20 mm, and the diameter of the target vessel was 2.25–5.0 mm. One lesion was selected at most, and one stent was implanted at most. The modified Rankin Scale (mRS) score was ≤ 3 before the last lesion, and there were at least 1 or more atherosclerotic plaque risk factors, including hypertension, diabetes, hyperlipidemia, hyperhomocysteinemia, or smoking history. The patients understood the purpose and procedure of the trial and voluntarily signed informed consent forms.

The key exclusion criteria were nonatherosclerotic arterial stenosis, intracranial hemorrhage or hemorrhagic cerebral infarction within 6 weeks, large-area cerebral infarction in stenosis-related areas (more than 1/3 of the vascular distribution area), cardiogenic embolism combined with a tumor, vascular malformation and aneurysm, allergy to the drugs and metal implants required for the study, clinical and imaging correlation analysis that could not determine that the target lesion was the responsible vessel, arteriography showing that the target lesions had severe calcification, intraluminal thrombosis, diffuse lesions, and tandem lesions.

### Randomization

All the patients who satisfied the inclusion criteria and did not have any of the exclusion criteria underwent a diagnostic cerebral angiogram before randomization, and the percent stenosis was measured using the WASID criteria (10). Patients meeting the clinical and angiographical criteria were randomly assigned 1:1 using a central stochastic method based on a computer system to either receive the Maurora Stent (DES group) or Apollo stent (BMS group). After filling in the randomization application form, the researcher logged into the randomization website for randomization. The computer system automatically assigned the randomization number and corresponding treatment group according to the patient's situation. After obtaining the randomization results, the researcher recorded the randomization number and corresponding stent information on the randomization application form and the original case and assigned the group according to the system (trial/control) to treat the patient. According to the characteristics of the tested products, this study could allow for patients to be blinded. All the groups underwent the stenting procedure within 48 h of randomization.

### Medical Therapy and Stenting Procedure

An Apollo stent (MicroPort Medical, Shanghai, China) was used for the DES group, and a Maurora stent (Alain Medical, Beijing, China) was used for the BMS group. Clopidogrel 75 mg and aspirin 300 mg were started 3–5 days before the operation. Nimodipine was given intravenously immediately before the operation. Systemic heparinization was given after a successful femoral/radial artery puncture. A 0.014-inch micro guide wire was selected to pass through the stenosis through the guide tube. According to the diameter of proximal and distal normal blood vessels, the stent was selected at a ratio of 1:1 or 0.9:1 according to the smaller diameter. The length of the stent was 4 mm longer than the length of the lesion (2 mm in the front and back). After the stent system was slowly passed into the narrow part, it was slowly expanded to the nominal pressure within 10 to 20 s according to the specified nominal pressure of the stent. After the stent was released, the balloon and micro guide wire were kept *in situ*, and the satisfactory fit between the stent and the blood vessel and the obvious leakage of contrast medium were immediately confirmed by the guided catheter angiography. If the stent fits well with the vessel wall and the vessel diameter returned to normal or the residual stenosis was <30%, the balloon was withdrawn slowly under DSA fluoroscopy. The blood pressure of the patients was controlled at 100–120/60–80 mmHg after the operation to prevent excessive perfusion. After pulling out the femoral artery sheath, low-molecular-weight (0.4 ml/12 h, 3 days) was injected subcutaneously. Aspirin 100–300 mg/day and clopidogrel 75 mg/day were taken orally until 3 months after the operation; clopidogrel was stopped after 3 months; and aspirin was reduced to 100 mg/day for long-term use.

### Follow-Up

Patients underwent postprocedural clinical and neurological evaluation at 24 h and on the day of discharge, including the NIHSS to assess neurological deficits and the mRS to assess neurological functional disability. An angiographic reexamination was performed 180 days after the operation to evaluate the in-stent restenosis rate. The angiographic reexamination was performed 180 days after the operation to evaluate the ISR rate. The clinical assessment and evaluation of neurological symptoms were performed by an NIHSS-certified study investigator who was not involved in the procedure. Follow-up visits occurred at 30 days, 180 days, and 1 year.

### Endpoints

The primary endpoint was the rate of in-stent restenosis at 180 days of randomization. The secondary endpoint was a composite of the following 2 outcomes: (1) ischemic stroke or transient cerebral ischemia (TIA) in the same territory as the presenting event (distal to the target lesion) between 30 days and 1 year of randomization; and (2) successful implantation of the stent. In-stent restenosis is defined as an in-stent stenosis rate at 180 days that is 30% higher than the postoperative residual stenosis rate (11). Procedure success was defined as stent success with no stroke or death before discharge.

### Safety Outcome Measures

The safety outcome was either stroke in any territory, death within 30 days of randomization, or adverse events. Adverse events refer to adverse medical events that happened during the clinical trial, regardless of whether they were related to the medical devices in the trial. The causes of adverse events may be related to the device, the drugs released on the device, the operation process, or the therapeutic drugs required in this protocol.

### Data and Safety Monitoring Board

A data and safety monitoring board (DSMB) composed of a neurointensivist, a vascular neurologist, an interventional neuroradiologist, and a biostatistician not otherwise involved with the study was responsible for overseeing the safety and ethical conduct of the trial.

### Statistical Analyses

Professional statisticians were responsible for formulating statistical analysis plans in consultation with the major researchers. The statistical analysis software adopted SAS® 9.4 software (software installation point authorization No.: 11202165). All the statistical tests adopted a two-sided test. If the *P*-value was ≤ 0.05, the tested difference was considered statistically significant. The description of quantitative indicators included the calculations of the mean, SD, median, minimum value, maximum value, lower quartile (Q1), and upper quartile (Q3). Categorical data are described as the number and percentage of each category. Group *t*-tests (homogeneity of variance and normal distribution) or Wilcoxon rank-sum tests were used for the intergroup comparison of quantitative data according to the data distribution. The chi-square test or exact probability method was used for classification data (if the chi-square test was not applicable). The Wilcoxon rank-sum test or CMH test was used for continuous data. When comparing the clinical outcomes, missing data were assumed to occur at random, and patients with missing data were excluded from the analysis.

### Sample Size Estimation

The sample size calculation software that was used was pass11. At the start of the trial, the statistical power to demonstrate a superior primary endpoint success rate for the DES group vs. the BMS group was anticipated to be ~90% with a total sample size of 156 patients and 1-sided α = 0.025 based on anticipated success rates of 5% and 19% in the DES group and BMS group, respectively (7, 12). Allowing for a combined 20% crossover, stent failure, withdrawal, and loss to follow-up rate, at least 188 participants needed to be enrolled.

## Results

### Baseline Characteristics

**Figure 1:**
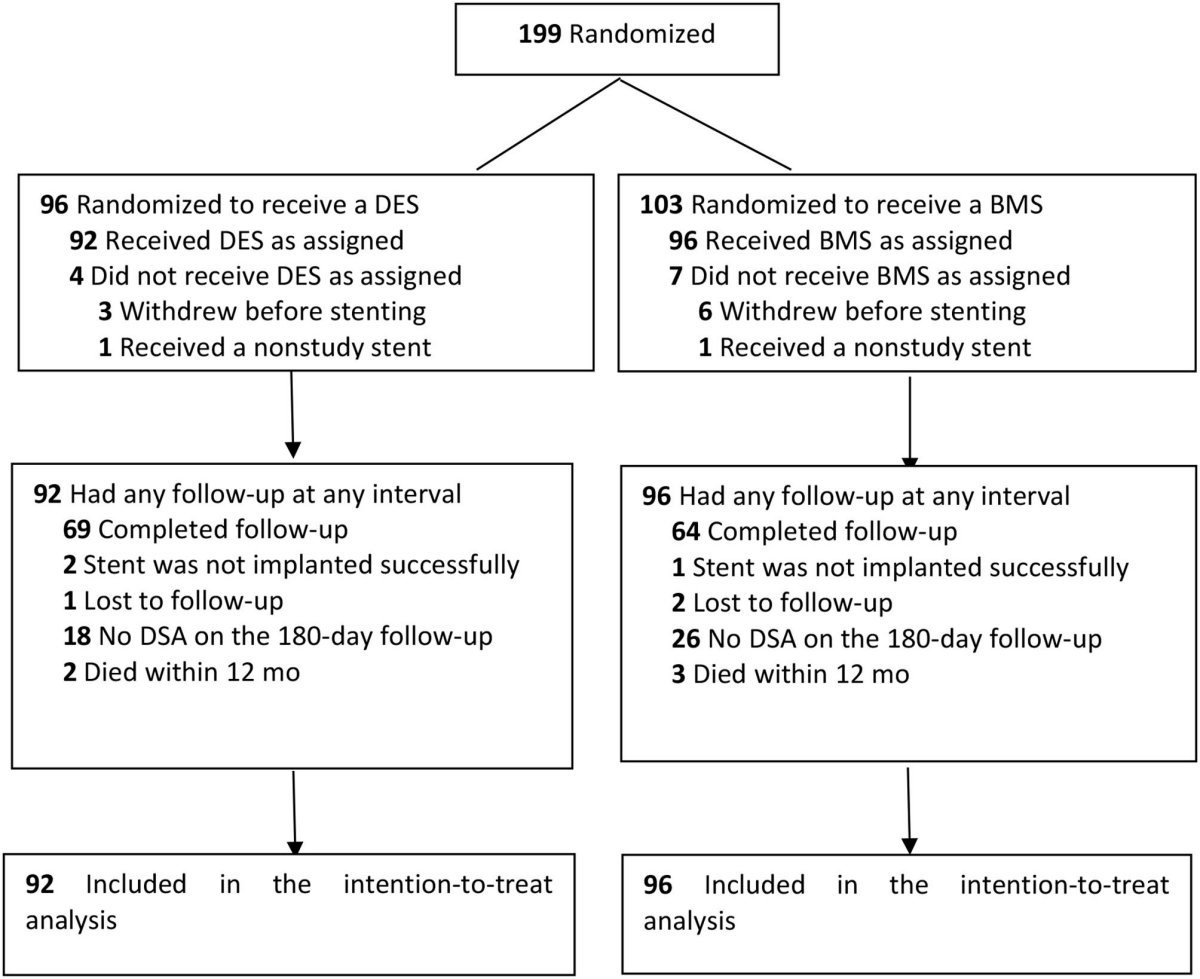
Design of the trial and flow of participants.

Between March 2014 and October 2015, a total of 199 patients underwent randomization in 10 centers. In total, nine patients (whose representatives withdrew consent after randomization) and two patients (who did not need stenting or had a nonstudy stent due to the condition after randomization) could not be included in the intention-to-treat analysis. A total of 133 patients completed the follow-up of the study protocol (69 in the DES group and 64 in the BMS group). The details are shown in Figure 1.

The mean age of the 188 study participants was 61.6 years (range, 38–75); 152 participants (80.9%) were men. The clinical risk factors for stroke, characteristics of the target artery, and mRS scores of prerandomization treatment were evenly distributed between the two treatment groups, as shown in Table 1.

**Table 1:** The baseline characteristics of the 188 patients.

**Characteristic**	**DES group (*N* = 92)^**a**^**	**BMS group (*N* = 96)^***a***^**
Age, mean (SD), year	61.7 (8.82)	61.5 (9.01)
Male sex	74 (80.0)	78 (81.3)
Weight, mean (SD), kg	70.2 (9.86)	72.6 (9.25)
Height, mean (SD), cm	167.9 (6.84)	168.0 (6.26)
Hypertension	69 (75.0)	71 (74.0)
**Blood pressure, mean (SD), mm Hg**		
Systolic	139.1 (18.04)	138.3 (16.27)
Diastolic	84.0 (10.51)	82.7 (12.41)
Hyperlipidemia	32 (34.8)	34 (35.4)
Diabetes mellitus	31 (33.7)	33 (34.4)
Coronary artery disease	21 (22.8)	17 (17.7)
Peripheral vascular disease	1 (1.1)	1 (1.0)
**Smoking history**		
Current	26 (28.3)	28 (29.2)
Former	27 (29.3)	32 (33.3)
Never	39 (42.4)	36 (37.5)
**Mori type**		
Mori A	61 (66.3)	58 (60.4)
Mori B	30 (32.6)	34 (35.4)
Mori C	1 (1.1)	4 (4.2)
**Arterial stenosis**		
Intracranial ICA	10 (10.9)	17 (17.7)
M1 middle cerebral artery segment	8 (8.7)	10 (10.4)
V1 vertebral artery segment	54 (58.7)	36 (37.5)
Intracranial vertebral artery	16 (17.4)	26 (27.1)
Basilar artery	4 (4.3)	7 (7.3)
Length of stenosis, mean (SD), mm	7.1 (3.40)	6.4 (2.85)
Percent stenosis of qualifying artery, mean (SD), %	82.7 (8.40)	84.3 (8.85)
mRS score, mean (SD)^b^	1.3 (0.70)	1.3 (0.60)

*ICA, Internal carotid artery; mRS, Modified Rankin's Disability scale.*

*^a^Data are reported as No. (%) unless otherwise indicated.*

*^b^The mRS score is based on a 7-point scale (0 indicates normal-6 indicates death)*.

### Primary Outcome

#### 180-Day Outcome

In total, there were 38 patients in the ITT population with in-stent restenosis at 180 days. Among these 38 patients with primary outcome events at the end of the 180-day follow-up period, 28 patients (43.8%) were in the BMS group, and 10 patients (14.5%) were in the DES group [risk difference, 29.3% (95% CI, 14.5%−44.0%); *P* = 0.001; Table 2].

**Table 2:** Primary and secondary endpoints in the intent-to-treat population.

	**DES group (*N* = 92)**	**BMS group (*N* = 96)**	**Difference (95% CI)**	***P*-Value**
**Primary end point at 180 days**				
In-stent restenosis	10/69 (14.5)	28/64 (43.8)	29.3 (14.5 to 44.0)	0.001
**Secondary end points**				
Ischemic stroke and TIA in the same territory as the presenting event (distal to the target lesion) between 30 days and 1 year of randomization	2/92 (2.2)	5/96 (5.2)	3.0 (−2.3 to 8.2)	0.354
Success rate of stent implantation	90/92 (97.8)	95/96 (99.0)	1.1 (−1.7 to 3.9)	0.584

*TIA, transient ischemic attack*.

### Secondary Outcomes

#### 1-Year Outcome

In the ITT analysis, the 1-year endpoint of ischemic stroke or TIA in the same territory between 30 days and 1 year was 5.2% (5/96) in the BMS group and 2.2% (2/92) in the DES group [risk difference, 3.0%; (95% CI, −2.3% to 8.2%); *P* = 0.354; Table 2].

The percent of patients having successful stent implantation was 99.0% (95/96) in the BMS group and 97.8% (90/92) in the DES group [risk difference, 1.1%; (95% CI, −1.7% to 3.9%); *P* = 0.584] (Table 2).

### Safety Outcomes

#### The 30-Day Outcome

Stroke in any territory and death at the 30-day follow-up occurred in five patients (5.2%) in the BMS group compared with three patients (3.3%) in the DES group [risk difference, 1.9%; (95% CI, −2.3% to 6.1%); *P* = 0.721; Table 3]. The 30-day all-cause mortality was 1 of 96 patients (1.0%) in the BMS group and one of 92 patients (1.1%) in the DES group (*P* = 1.000; Table 3). The two deaths were all related to hemorrhagic stroke (3 days after the stent procedure in the BMS group and 2 days after the procedure in the DES group). In total, one death was related to myocardial infarction 10 months after surgery, and one death was related to malignancy 5 months after surgery in the BMS group. One death occurred in the DES group 4 months after surgery, and this death was related to acute lymphoblastic leukemia that was not diagnosed before randomization.

**Table 3:** Safety endpoints in the intent-to-treat population.

	**DES group (*****N*** **=** **92)**		**BMS group (*****N*** **=** **96)**		**Difference (95% CI)**	***P*-Value**
	**Person times**	**Rate**	**Person times**	**Rate**		
Stroke in any territory and death within 30-day of randomization	3	3/92 (3.3)	5	5/96 (5.2)	1.9 (−2.3 to 6.1)	0.721
Death	1	1/92 (1.1)	1	1/96 (1.0)		1.000
Total adverse events	114	59/92 (64.1)	167	72/96 (75.0)	10.9 (−0.1 to 21.7)	0.115
Mild adverse events	87	53/92 (57.6)	121	61/96 (63.5)	5.9 (−0.4 to 12.3)	0.456
Related to the test device	10	10/92 (10.9)	17	16/96 (16.7)	5.8 (−1.7 to 10.0)	0.294
Related to the operation	17	14/92 (15.2)	13	11/96 (11.5)	−3.7 (−8.9 to 1.4)	0.522
Medium adverse events	13	11/92 (12.0)	19	16/96 (16.7)	4.7 (−0.8 to 10.2)	0.409
Related to the test device	3	3/92 (3.3)	4	4/96 (4.2)	0.9 (−1.1 to 2.9)	1.000
Related to the operation	1	1/92 (1.1)	2	2/96 (2.1)	1.0 (−0.8 to 2.9)	1.000
Serious adverse events	14	10/92 (10.9)	27	23/96 (24.0)	13.1 (5.0 to 21.3)	0.022
Related to the test device	1	1/92 (1.1)	11	9/96 (9.4)	8.3 (1.5 to 15.0)	0.019
Related to the operation	0	0	2	2/96 (2.1)	2.1 (−0.2 to 4.4)	0.498

#### Adverse Events

The total number of adverse events was 167 in 72 patients (75.0%) in the BMS group compared with 114 in 59 patients (64.1%) in the DES group [risk difference, 10.9%; (95% CI, −0.1% to 21.7%); *P* = 0.115]. In total, 27 serious adverse events occurred in 23 patients (24.0%) in the BMS group compared with 14 events in 10 patients (10.9%) in the DES group [risk difference, 13.1%; (95% CI, 5.0% to 21.3%); *P* = 0.022]. Serious adverse events related to the test device occurred 11 times in nine patients (9.4%) in the BMS group compared with one time in one patient (1.1%) in the DES group [risk difference, 8.3%; (95% CI, 1.5%−15.0%); *P* = 0.019]. The details are shown in Table 3.

A total of 23 organ systems participated in the adverse events in this trial. There were 35 nervous system adverse events that occurred in 30 patients (31.3%) in the BMS group compared with 23 events in 22 patients (23.9%) in the DES group [risk difference, 7.3% (95% CI, −1.7% to 17.3%); *P* = 0.721]. General diseases and various reactions at the administration site occurred in 26 patients (27.1%) in the BMS group compared with 11 patients (12.0%) in the DES group [risk difference, 15.1%; (95% CI, 6.4% to 23.7%); *P* = 0.01]. The details are shown in Table 4.

**Table 4:** The different diseases and medical codes in the adverse event record.

	**DES group (*****N*** **=** **92)**		**BMS group (*****N*** **=** **96)**		**Difference (95% CI)**	***P*-Value**
	**Person times**	**Rate**	**Person times**	**Rate**		
Nervous system diseases	23	22/92 (23.9)	35	30/96 (31.3)	7.3 (−1.7 to 17.3)	0.721
General diseases and various reactions at the administration site	12	11/92 (12.0)	28	26/96 (27.1)	15.1 (6.4 to 23.7)	0.01
Heart disease	8	8/92 (8.7)	15	11/96 (11.5)	2.8 (−1.2 to 6.8)	0.631
Various injuries, poisoning and surgical complications	12	9/92 (9.8)	13	12/96 (12.5)	2.7 (−1.6 to 6.9)	0.646
Infectious and infectious diseases	8	7/92 (7.6)	12	9/96 (9.4)	1.8 (−1.7 to 5.3)	0.796
Vascular and lymphatic diseases	4	4/92 (4.4)	12	11/96 (11.5)	7.1 (−4.8 to 19.0)	0.105
Diseases of the blood and lymphatic system	11	11/92 (12.0)	10	9/96 (9.4)	−2.6 (−7.2 to 2.1)	0.640
Gastrointestinal diseases	7	7/92 (7.6)	9	7/96 (7.3)	−0.3 (−0.9 to 0.3)	1.000
Metabolic and nutritional diseases	5	4/92 (4.4)	6	5/96 (5.2)	0.9 (−0.2 to 2.0)	1.000
Respiratory, thoracic and mediastinal diseases	3	3/92 (3.3)	5	5/96 (5.2)	1.9 (−2.3 to 6.1)	0.721
Kidney and urinary diseases	6	6/92 (6.5)	3	3/96 (3.1)	−3.4 (−8.4 to 1.6)	0.323
Eye diseases	6	4/92 (4.3)	3	3/96 (3.1)	−1.2 (−3.1 to 0.7)	0.716
Hepatobiliary diseases	1	1/92 (1.1)	3	2/96 (2.1)	1.0 (−0.8 to 2.9)	1.000
Musculoskeletal and connective tissue diseases	1	1/92 (1.1)	3	3/96 (3.1)	2.0 (−1.1 to 5.1)	0.621
Endocrine system diseases	1	1/92 (1.1)	2	2/96 (2.1)	1.0 (−0.8 to 2.9)	1.000
Ear and labyrinth diseases	0	0	0	2/96 (2.1)	2.1 (−0.2 to 4.4)	0.498
Immune system diseases	2	2/92 (2.2)	1	1/96 (1.0)	−1.1 (−2.6 to 0.4)	0.615
Mental disease	1	1/92 (1.1)	1	1/96 (1.0)		1.000
Benign, malignant and unknown tumors	1	1/92 (1.1)	1	1/96 (1.0)		1.000
Skin and subcutaneous diseases	1	1/92 (1.1)	1	1/96 (1.0)		1.000
Various medical examinations	0	0	0	1/96 (1.0)	1.0 (−0.4 to 2.4)	1.000
Various surgical and medical operations	0	0	0	1/96 (1.0)	1.0 (−0.4 to 2.4)	1.000
Reproductive system and breast diseases	1	1/92 (1.1)	0	0	−1.1 (−2.4 to 0.2)	0.489

The criteria for determining the severity of adverse events are as follows: mild: the symptoms are mild, do not affect the patient's normal activities, or the symptoms are transient, do not need treatment, and there are no sequelae; medium: affect the patient's normal activities; serious: the patient has fatal or immediately life-threatening clinical symptoms and needs hospitalization; or there is a disability that may endanger the patient's life or the patient may lose the ability for daily living.

## Discussion

To our knowledge, this is the first randomized, multicenter, prospective, drug-eluting stent trial for symptomatic intracranial stenosis and vertebral artery stenosis. Although it differed in its design and the type of stent used, this study showed similar results to some previous clinical trials (2, 3, 5–9). In the current trial, a lower rate of ISR was shown with DES than with BMS in symptomatic intracranial arterial stenosis and vertebral artery stenosis.

The present trial demonstrated a higher than expected rate of ISR in both the DES and BMS groups: 14.5% at the 180-day follow-up in the DES group vs. 43.8% in the BMS group, with an absolute difference of 29.3%. The ISR of the DES group was 14.5%, which was higher than the 5% incidence that was predicted by the application of a coronary drug-eluting stent in cerebrovascular treatment reported according to relevant literature at the time of trial design (7). However, there have also been reports of a higher ISR rate. In a former study, Fields et al. (13) reported the insertion of a DES with cerebrovascular stenosis in 27 patients. The angiographic 180-day follow-up showed that the ISR rate was 22% (13). The ISR in the BMS group was 43.8%, which was also higher than that reported in some clinical studies. In the SSYLVIA balloon-mounted intracranial stenosis study, 43 lesions were treated, and the ISR rate (50% higher than the postoperative residual stenosis rate) was as high as 32.4% at the 180-day follow-up (14). The ISR of our study was also higher than that in Jin's study, which showed that the ISR with the Apollo stent was 27.5% (24/87) vs. the Wingspan, which was 24.6% (17/69) (15). The higher ISR of our study may, in part, be related to the study design, in which the ISR was defined as the stenosis rate at 180 days being 30% higher than the postoperative residual stenosis rate, rather than at 50%.

The incidence of ischemic stroke or TIA in the same territory between 30 days and 1 year was 5.2% in the BMS group and 2.2% in the DES group. The incidence of stroke in any territory and death at the 30-day follow-up were 5.2% in the BMS group and 3.3% in the DES group. Our results are similar to those of some studies on the use of DESs and BMSs in the cerebrovasculature. In the SSYLVIA trial, four patients (6.6%) had strokes, and no deaths occurred in the first 30 days, with four of 55 patients (7.3%) having strokes later than 30 days and one of which was the only patient not stented (14). The Wingspan One-Year Vascular Events and Neurologic Outcomes (WOVEN) trial consequently studied the WEAVE trial and was presented at the 2020 ISC. In the 1-year follow-up period, there were 11 strokes or deaths of the 129 patients (8.5%) at the 1-year follow-up (16). In the other two similar trials, the 30-day event rates were 6.5% and 11.0% (17, 18). The technical success rate was 99.0% (95/96) in the BMS group and 97.8% (90/92) in the DES group. All the three unsuccessful cases were because the lesion was located in the MCA and the vessel was too tortuous, resulting in the stent being unable to reach the target lesion. Our results are similar to the 95% that was reported in the SSYLVIA trial and was better than the 79% that was reported in the VISSIT trial (14, 19). A retrospective study using the same Apollo stent to examine 92 patients with symptomatic intracranial stenosis showed a deployment success rate of 98.9% (18). However, these studies are not comparable to this trial. These studies had a different study design, population, and stent type. Moreover, the majority were based on self-reported data and lacked independent raters or event adjudicators (20, 21).

The adverse events and their correlation with the instruments and surgery in this study were analyzed based on the evaluation results of the independent safety committee to reduce bias of the analysis. Overall, the incidence of adverse events was similar in the DES group and the BMS group. Among the adverse events in this trial, 23 organ systems were involved. The most common adverse events were various nervous system diseases (23.91% in the DES group vs. 31.25% in the BMS group).

### Limitation

Finally, some limitations also exist in our study. The current trial was not double-blinded due to the lack of feasibility of masking for the type of stents used, which might cause some bias. In the current trial, the experience of using the Maurora stent in the DES group was less than that of using the Apollo stent in the BMS group. Second, the current trial did not deliberate on segmenting the anterior and posterior circulation patients separately. Because the natural history of anterior and posterior circulation atherosclerosis is different, the technical operation details, clinical prognosis and in stent restenosis rate of stent implantation are also different. Third, the study was performed in China; therefore, the findings could not be generalized to other ethnic groups. Fourth, the sample size in our study was relatively small, and the numbers of participants from each of the sites were quite different.

## Conclusion

Among the patients with symptomatic intracranial arterial stenosis and vertebral artery stenosis, the use of a drug-eluting stent compared with a bare metal stent resulted in a decreased risk of ISR, a similar rate of successful implantation of the stent, and similar adverse events. These findings support the use of a drug-eluting stent for patients with symptomatic intracranial arterial stenosis and vertebral artery stenosis.

## Data Availability Statement

The original contributions presented in the study are included in the article/supplementary material, further inquiries can be directed to the corresponding author/s.

## Ethics Statement

The studies involving human participants were reviewed and approved by Ethics Committee of Beijing Tiantan Hospital. The patients/participants provided their written informed consent to participate in this study.

## Author Contributions

J-HS: study design, literature search, data collection, database establishment, and chief writer of this manuscript. NM, FG, and D-PM: data analysis. GL: manuscript reviewing, modification, and data analysis. Z-RM: study design, chief writer of this manuscript, and guarantor. All authors contributed to the article and approved the submitted version.

## Funding

This study was supported by the National Natural Science Foundation of China (81371290) and Beijing High-level Personnel Funds (2013-2-019). This study was also funded by the National Science and Technology Support Program of the 12th Five-Year Plan of the Ministry of Science and Technology (2011BAI08B02).

## Conflict of Interest

The authors declare that the research was conducted in the absence of any commercial or financial relationships that could be construed as a potential conflict of interest. The reviewer XG declared a shared parent affiliation with the authors to the handling editor at the time of review.

## Publisher's Note

All claims expressed in this article are solely those of the authors and do not necessarily represent those of their affiliated organizations, or those of the publisher, the editors and the reviewers. Any product that may be evaluated in this article, or claim that may be made by its manufacturer, is not guaranteed or endorsed by the publisher.
